# Interfacial Engineering of Ti_3_C_2_T_*x*_ MXene Electrode Using g-C_3_N_4_ Nanosheets for High-Performance Supercapacitor in Neutral Electrolyte

**DOI:** 10.1021/acsomega.4c01353

**Published:** 2024-05-06

**Authors:** Manopat Depijan, Kanit Hantanasirisakul, Pasit Pakawatpanurut

**Affiliations:** † Department of Chemistry, Center of Excellence for Innovation in Chemistry, and Center of Sustainable Energy and Green Materials, Faculty of Science, Mahidol University, 272 Rama VI Road, Ratchathewi, Bangkok 10400, Thailand; ‡ Centre of Excellence for Energy Storage Technology (CEST), Department of Chemical and Biomolecular Engineering, School of Energy Science and Engineering, Vidyasirimedhi Institute of Science and Technology, Wangchan Valley, Rayong 21210, Thailand

## Abstract

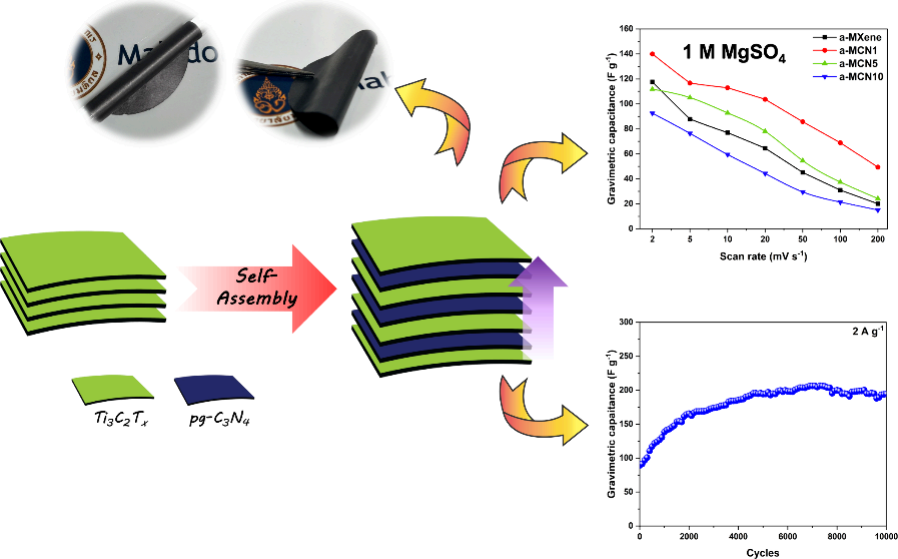

The superior performance of the Ti_3_C_2_T_*x*_ (MXene)-based supercapacitor in acidic electrolytes has recently gained much interest in the energy storage community. Nevertheless, its performance in most neutral electrolytes is unfavorably low, plausibly due to limited ion diffusion between the MXene layers. Herein, protonated g-C_3_N_4_ (pg-C_3_N_4_) is incorporated into the Ti_3_C_2_T_*x*_ electrode by using a facile self-assembling process and annealing, which results in increased interlayer *d*-spacing and electrical conductivity of the composite electrode. As a result, the annealed Ti_3_C_2_T_*x*_/pg-C_3_N_4_ film revealed an enhanced ion-accessibility and gravimetric capacitance of 140 F g^–1^ in 1 M aqueous MgSO_4_ electrolyte. The cyclic stability test also indicates excellent capacitance retention, with negligible loss of capacitance over 10000 cycles.

## 1 Introduction

Carbon-based supercapacitors (SCs) have become one of the most commercialized energy storage systems^1^ due to their high-power density, low cost, inertness to electrochemical reactions, and long life cycle unmatched by batteries and other energy storage systems.^2,3^ Furthermore, the structures of SCs can also be made either rigid or flexible, which accommodates increasing demand for wide-ranging applications that include modern portable devices.^4,5^ However, the energy density delivered by current carbon-based SCs is still limited, despite several improvements made.^6−10^

Recently, two-dimensional transition metal carbides, known collectively as MXenes, have emerged as a novel two-dimensional material widely developed for various applications, especially in the energy storage field.^11,12^ MXene has a general formula of M_*n*+1_X_*n*_T_*x*_, where M, X, and T_*x*_ stand for early transition metals, carbon and/or nitrogen, and the surface terminations, respectively.^13^ Among a number of materials within the MXene family, Ti_3_C_2_T_*x*_ is perhaps one of the most studied due to its many advantages, which include high electrical conductivity, abundant surface functionalities, reasonable chemical stability, and environmental friendliness.^14−16^ In acidic electrolytes, the Ti_3_C_2_T_*x*_ electrode was reported to achieve an outstanding capacitance of up to 1500 F cm^–3^ or 370 F g^–1^, which was much higher than that of the carbon-based supercapacitors.^17^ Additionally, neutral electrolytes were able to expand the potential window that boosted the energy density.^15,18^ Nonetheless, Ti_3_C_2_T_*x*_ in neutral electrolytes reached a maximum capacitance of only 300 F cm^–3^ or 97 F g^–1^.^15,19−22^ The limited capacitance of neutral electrolytes can be ascribed to the larger hydrated ion size, lower ionic conductivity, and slower ion diffusion efficiency than acidic electrolytes.^20,23,24^

Many strategies have been proposed to resolve the above-mentioned issue. Organic molecule exfoliation utilized large molecular size to expand interlayer *d*-spacing during MXene synthesis. However, this technique resulted in lower electrical conductivity and a smaller flake size of Ti_3_C_2_T_*x*_.^25−27^ Meanwhile, the heteroatom doping technique also showed effective interlayer spacing and material surface enhancement.^28^ However, this technique commonly requires high-temperature conditions, which could potentially degrade MXene sheets and is not environmentally friendly.^28,29^ Lastly, heterostructure compositing has also been found to enlarge *d*-spacing and provide facile synthesis protocol.^30,31^ For example, a carbon nanotube decorated on Ti_3_C_2_T_*x*_ via a simple self-assembly process indicated an enlargement in the interlayer spacing. This pillaring effect provided an improved capacitance of up to 390 F cm^–3^ or 150 F g^–1^ in 1 M MgSO_4_ electrolyte.^32^ Similarly, Fe_2_O_3_ nanoparticle as a dopant in Ti_3_C_2_T_*x*_ was found to increase the *d*-spacing and provide an outstanding capacitance of 2607 F cm^–3^ or 583 F g^–1^.^33^

Graphitic carbon nitride (g-C_3_N_4_) has received attention in energy storage and conversion applications due to its simple synthesis procedure, low cost, high abundance, excellent thermal and chemical stability, and environmental friendliness.^34−36^ Even though g-C_3_N_4_ is a semiconductor, the tri-*s*-triazine and triazine can create an ion-accessible and transportation channel, yielding relatively high energy storage capability.^37,38^ For instance, Lu et al. deposited g-C_3_N_4_ on reduced graphene oxide (rGO) via an in situ growing technique.^37^ The results indicated a high energy density of about 281.3 μW h cm^–2^ at 1 mA cm^–2^ that was affected by enhanced electrical conductivity and ion-accessible surface area.^37^ Similarly, Lin et al. prepared g-C_3_N_4_/graphene oxide (GO) aerogels by a hydrothermal process.^39^ This composite indicated a high specific capacitance of about 170.7 F g^–1^ and as high an energy density as 7.47 Wh kg^–1^ in an acidic electrolyte.^39^ Recently, the supercapacitor made of g-C_3_N_4_/Ti_3_C_2_T_*x*_ composite was reported by Xu et al. with specific capacitance as high as 552 F g^–1^ in H_2_SO_4_, which was about 3 times higher than that of the pristine Ti_3_C_2_T_*x*_.^40^ Furthermore, Zhang and colleagues developed a flexible solid-state g-C_3_N_4_/Ti_3_C_2_T_*x*_ supercapacitor, achieving an energy density of 23.98 Wh kg^–1^ with a power density of 139.66 W kg^–1^.^41^ Notably, the composite film maintained its flexibility and retained good performance even under bending conditions.^41^ These results underscore the capacity of g-C_3_N_4_ to augment the performance of Ti_3_C_2_T_*x*_, despite its inherent high electrical resistance.

Herein, the protonated g-C_3_N_4_ (pg-C_3_N_4_)-doped Ti_3_C_2_T_*x*_ freestanding electrodes were fabricated through a facile self-assembling process. The pg-C_3_N_4_ content was varied from 1 to 10 wt % with respect to Ti_3_C_2_T_*x*_. The addition of 1 wt % pg-C_3_N_4_ was found to induce the enlargement of the *d*-spacing of Ti_3_C_2_T_*x*_, with significant increase in the overall electrical conductivity, resulting in improved electrochemical performance of the composite electrodes. To further increase the electrical conductivity, the composite was subjected to thermal annealing, resulting in better surface contact between pg-C_3_N_4_ and Ti_3_C_2_T_*x*_ via O-terminated sites and enhanced electrical conductivity. The stable potential window was found to be 1.1 V in 1 M MgSO_4_. The 1 wt % Ti_3_C_2_T_*x*_/pg-C_3_N_4_ composite electrode not only showed the highest gravimetric capacitance of 140 F g^–1^ but also indicated excellent capacitance retention over 10000 cycles.

## 2 Results and Discussion

### 2.1 Freestanding Film of Ti_3_C_2_T_*x*_/pg-C_3_N_4_

In this work, the Ti_3_C_2_T_*x*_ nanosheet was synthesized using the minimally intensive layer delamination (MILD) method. In this method, hydrofluoric acid was formed *in situ* from the reaction between lithium fluoride and hydrochloric acid, and the lithium intercalation between the MXene layer provided one-step exfoliation of Ti_3_C_2_T_*x*_.^42^ The XRD diffractogram showed a shift in the (002) plane toward lower angle after the etching process (Figure S1a), suggesting the expansion of the *d*-spacing expanded from 9.28 to 12.36 Å. The UV–vis spectrum showed a single absorption at 760 nm (Figure S1b), and the AFM imaging revealed a single layer of Ti_3_C_2_T_*x*_ with a thickness of about 1.56 nm (Figure S1c). This data indicated a successful synthesis of the Ti_3_C_2_T_*x*_ nanosheet, consistent with the previous literature.^27,42,43^

The graphitic carbon nitride (g-C_3_N_4_) was synthesized via thermal polymerization of dicyanamide, using ammonium chloride as a pre-exfoliating agent.^44^ The resulting g-C_3_N_4_ powder obtained from this method showed a paler yellow color when compared to g-C_3_N_4_ prepared without using ammonium chloride (Figure S2a), suggesting thinner sheets of g-C_3_N_4_.^44^ The obtained g-C_3_N_4_ powder was further exfoliated and protonated using concentrated H_2_SO_4_. This procedure generates rapid heat via an exothermic reaction between strong acid and water molecules. Consequently, hydrogen bonds and the NH– linkers in each layer were dissociated, producing ultrathin nanosheets.^45,46^ The XRD analysis revealed the disappearance of all peaks after protonation except the one at 27.62° (Figure S2b), which indicates complete g-C_3_N_4_ nanosheet exfoliation.^47^ Infrared spectroscopy was carried out to study the surface functional groups of g-C_3_N_4_. The peaks located at about 1543 and 803 cm^–1^ corresponding to C=N stretching and out-of-plane tri-s-triazine bending. After protonation, these two peaks almost completely disappeared, which suggests the formation of the protonated form of g-C_3_N_4_ (Figure S2c).^45,46,48^ In addition, the particle size distribution analysis showed a particle size of pg-C_3_N_4_ smaller than that of g-C_3_N_4_ (Figure S3), which was also observed via the top-view SEM image (Figure S4). These data together confirmed the formation of the pg-C_3_N_4_ nanosheets.

**Scheme 1:**
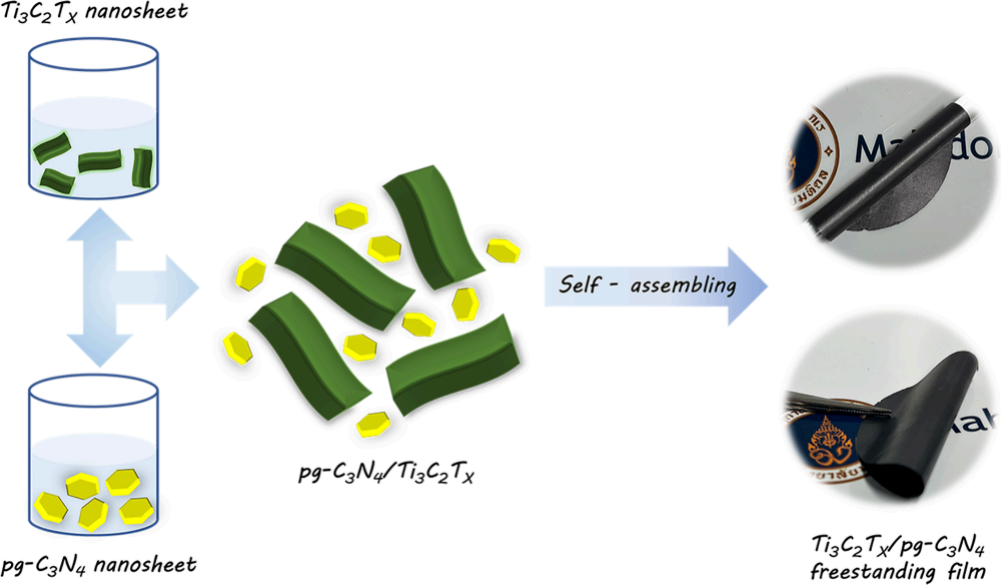
Schematic Illustration of the Fabrication of Ti_3_C_2_T_*x*_/pg-C_3_N_4_ Heterostructure via the Self-Assembling Process

Based on the zeta potentials of −37.9 mV for Ti_3_C_2_T_*x*_ and +18.3 mV for pg-C_3_N_4_, the self-assembly strategy was employed to prepare the composite film of the two materials. After the suspensions of pg-C_3_N_4_ and Ti_3_C_2_T_*x*_ were mixed, agglomerated particles of Ti_3_C_2_T_*x*_/pg-C_3_N_4_ were observed (Figure S5a). The precipitates were then vacuum-filtrated, resulting in flexible free-standing films of Ti_3_C_2_T_*x*_/pg-C_3_N_4_ (Scheme 1). All of the freestanding composite films appeared homogeneous, without any ruptures (Figure S5b,c).

**Figure 1:**
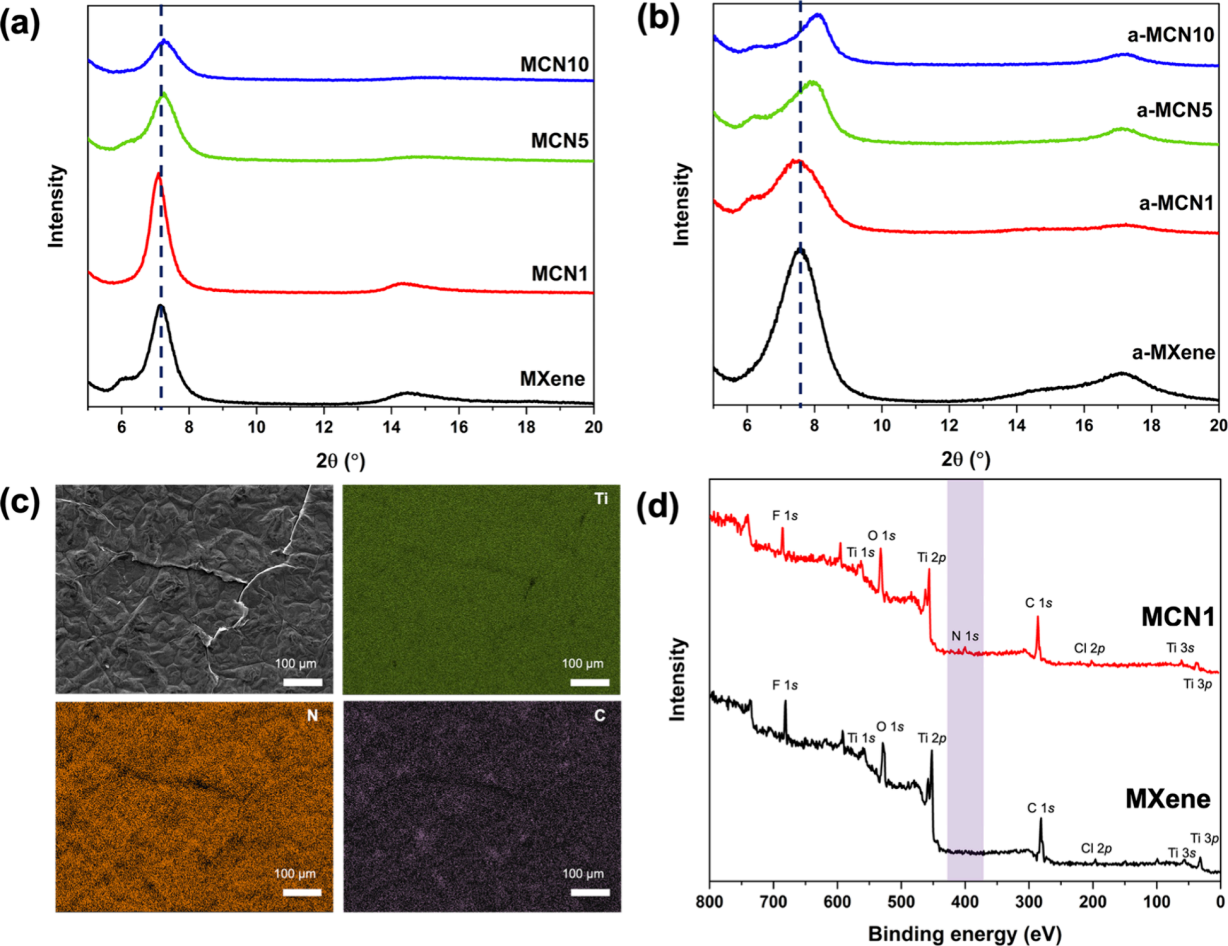
(a) XRD diffractograms of the freestanding composite films before annealing and (b) after annealing at 200 °C for 2 h under Ar, (c) top-view SEM image and the element mapping analysis of MCN1, and (d) comparison of the XPS survey between MXene and MCN1.

The top-view element mapping analysis of 1 wt % pg-C_3_N_4_-doped Ti_3_C_2_T_*x*_ (MCN1) revealed that well-distributed N element well aligned with Ti and C elements (Figure 1c). The XPS survey spectra also showed the presence of N atoms even at 1 wt % doping of pg-C_3_N_4_ (Figure 1d), suggesting successful anchoring of pg-C_3_N_4_ into Ti_3_C_2_T_*x*_. The cross-sectional SEM image revealed compact layers of all films, which were characteristic of Ti_3_C_2_T_*x*_ (Figure S6).

The interlayer spacing of the freestanding films was assessed by using the (002) peak in the X-ray diffractograms. According to the *d*-spacing of the freestanding film calculated and summarized in Table 1, MCN1 illustrated an enlargement in *d*-spacing when compared to the pristine MXene. However, we found that an excessive amount of pg-C_3_N_4_ could result in a decrease in *d*-spacing as well as lower crystallinity, as observed for MCN5 and MCN10 (Figure 1a). The electrical conductivity of the freestanding films was obtained using a 4-point probe measurement (see Table 1). Introducing pg-C_3_N_4_ was found to improve the electrical conductivity, which was plausibly due to the generation of conjugated network between pg-C_3_N_4_ nanosheets and Ti_3_C_2_T_*x*_.^37,48^ In the case of MCN10, the large increase in electrical conductivity could be attributed to the lowing in *d*-spacing, resulting in shortened conductive pathway.^49,50^

**Table 1:** Physical Properties and Electrochemical Performance of Freestanding Composite Films

sample	*d*-spacing (Å)	electrical conductivity (S cm^–1^)	gravimetric capacitance (F g^–1^) @2 mV s^–1^	areal capacitance (mF cm^–2^) @2 mV s^–1^	rate performance (%)
MXene	12.36	7212	84	78	21.1
MCN1	12.46	7363	119	90	26.1
MCN5	12.17	7554	110	88	20.6
MCN10	12.12	9014	83	74	16.1
a-MXene	11.38	10411	118	84	17.1
a-MCN1	11.70	8223	140	93	35.3
a-MCN5	11.07	6765	112	85	21.7
a-MCN10	10.93	6024	93	70	16.3

**Figure 2:**
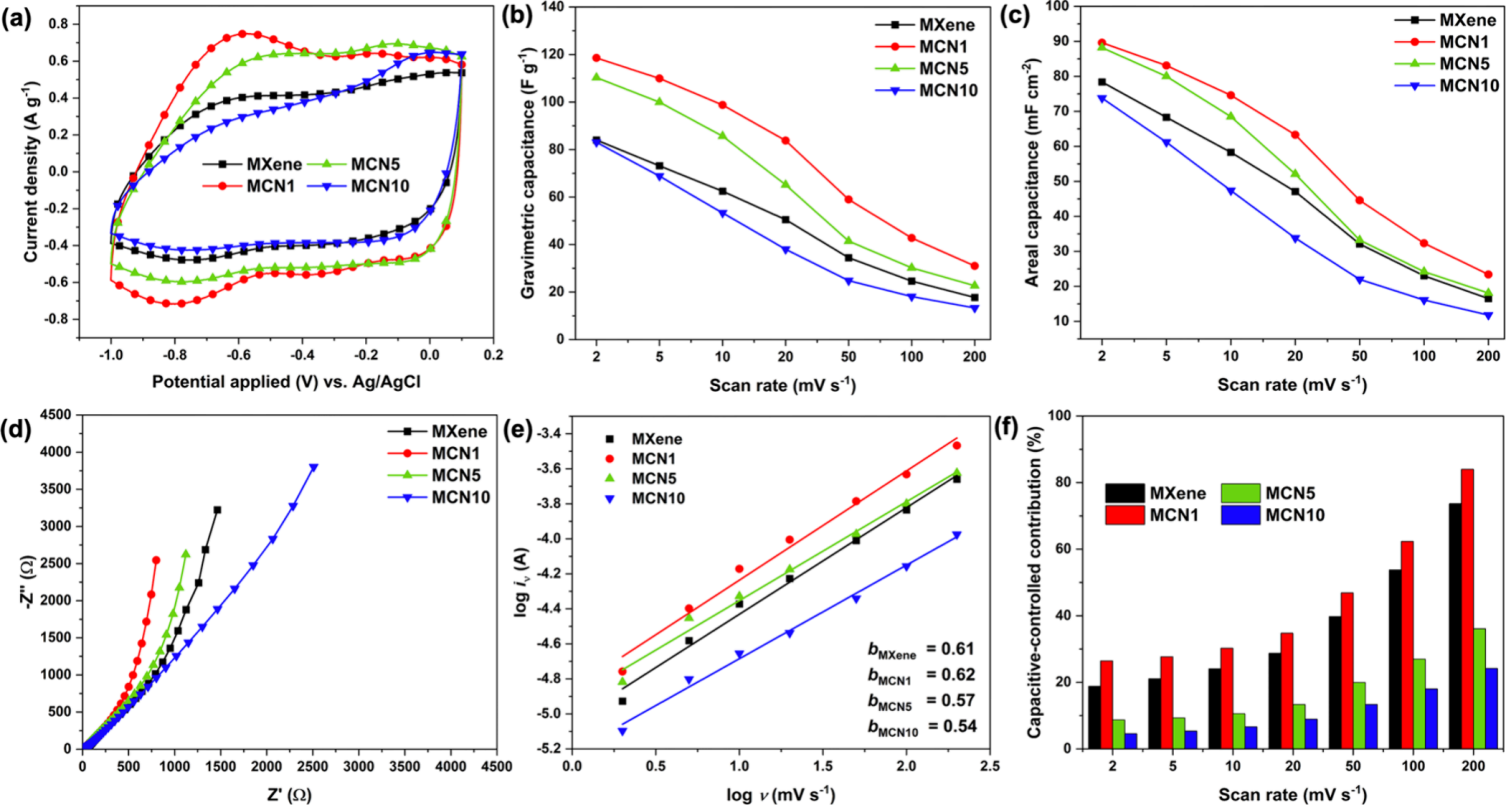
Electrochemical characterization of the Ti_3_C_2_T_*x*_/pg-C_3_N_4_: (a) Cyclic voltammogram of the freestanding films at 5 mV s^–1^, (b) calculated gravimetric and (c) areal capacitances, (d) EIS spectra, (e) *b* values obtained from the relationship between the anodic peak current and potential scan rate, and (f) comparison of capacitive contribution obtained from the Dunn’s method fitting.

To demonstrate the performance of the Ti_3_C_2_T_*x*_/pg-C_3_N_4_ composite film, electrochemical analysis was performed in 1 M MgSO_4_. MgSO_4_ is one of the neutral electrolytes that is cheap and a harmless chemical. However, its low ionic conductivity affects the rate performance. Therefore, the improvement of this issue depends rather on electrode material and structure.^19,32^ In a three-electrode system, the composite films provided a stable potential window from 0.1 to −1.0 V without electrolysis. Compared to other reports, this potential window was wider, which might be due to the higher electrical conductivity and unique electronic structure of the MILD-synthesized Ti_3_C_2_T_*x*_.^26,51−54^ The obtained cyclic voltammogram (CV) profiles indicated two humps at −0.65 and −0.75 V, which can be attributed to the two-stage intercalation of Mg^2+^ ions.^19^ All of the CV profiles exhibited a quasi-rectangular shape. MCN1 and MCN5 illustrated the improvement of intercalation signal at −0.65 V when compared to the pristine MXene, while this signal was dramatically decreased for MCN10 (Figure 2a). The gravimetric capacitance (Figure 2b) and areal capacitance (Figure 2c) were calculated in various scan rates ranging from 2 to 200 mV s^–1^. At the lowest scan rate, MCN1 achieved the highest gravimetric capacitance and areal capacitance of about 119 F g^–1^ and 89.6 mF cm^–2^, respectively, followed by MCN5 and MCN10. At the scan rate of 200 mV s^–1^, the retained gravimetric capacitance compared to the initial scan rate was calculated to be 21.1, 26.1, 20.6, and 16.0% for MXene, MCN1, MCN5, and MCN10, respectively. The internal resistance of the electrode was then studied by using electrochemical impedance spectroscopy (EIS) according to the equivalent circuit model in Figure S7. The overall resistance determined from the EIS analysis revealed that MCN1 and MCN5 possessed lower resistance than that of MXene (Figure 2d). The charge-transfer resistances of the freestanding films were determined to be 50.3, 29.4, 33.5, and 385.0 Ω for MXene, MCN1, MCN5, and MCN10, respectively. As the pg-C_3_N_4_ content was increased, the crystallinity and *d*-spacing were lower, resulting in limited ion diffusion and higher charge-transfer resistance, as well as lower rate performance.^55,56^ Moreover, the excess amount of pg-C_3_N_4_ could induce a bulk structure of g-C_3_N_4_, potentially impeding charge storage and diffusion.^37^

To further understand about the charge storage behavior, the kinetic information was investigated using a relationship between current response and potential scan rate based on eq 1, where *i*_(ν)_, ν, and *a* and *b* are the current under measured sweep rate, scan rate (mV s^–1^), and adjustable parameters, respectively.^57,58^ According to Figure 2e, the *b* value was measured to be 0.61, 0.62, 0.56, and 0.54 for MXene, MCN1, MCN5, and MCN10, respectively. In comparison, the quantitative analysis to differentiate charge contribution was performed using the Dunn’s method (eq 2), where *k*_1_ and *k*_2_ denote capacitive-controlled and diffusion-controlled constants, respectively. MCN1 has a slightly higher capacitive contribution than MXene for all of the scan rates, whereas MCN5 and MCN10 demonstrated a diffusion-controlled contribution (Figure 2f). MCN1 indicated a more pronounced surface capacitive contribution as a result of greater electrical conductivity without a significant change in crystallinity as pg-C_3_N_4_ was introduced into the structure. This result was also consistent with the CV profiles, at which the intercalation signal was increased for MCN1.
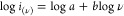
1
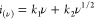
2

Therefore, doping MXene with 1 wt % pg-C_3_N_4_ resulted in an optimal composite that showed enhanced capacitance and rate performance. The interaction between Ti_3_C_2_T_*x*_ and pg-C_3_N_4_ could be further reinforced by an annealing post-treatment process, which was expected to provide improved gravimetric capacitance and rate performance.

### 2.2 Effect of Annealing

The annealing process was performed at 200 °C under an Ar atmosphere. At this temperature, water molecules within the freestanding films are removed, generating higher electrical conductivity.^14^ Moreover, the annealing process could create stronger interaction between pg-C_3_N_4_ and Ti_3_C_2_T_*x*_ that was able to improve the ion-accessibility within the composite.^48^ The electrical conductivity of the annealed freestanding films was measured to be 10411, 8223, 6765, and 6024 S cm^–1^ for a-MXene, a-MCN1, a-MCN5, and a-MCN10, respectively. Interestingly, a-MCN5 and a-MCN10 displayed lower electrical conductivity when compared to the other samples, annealed, and unannealed. The reduction in electrical conductivity could be due to the reaggregation of pg-C_3_N_4_ after the thermal treatment, which induced a bulk formation of pg-C_3_N_4_. This hypothesis was demonstrated by heating a pg-C_3_N_4_ suspension at 200 °C for 2 h, which resulted in precipitation of pg-C_3_N_4_ (Figure S8). The calculated *d*-spacing was reduced for all composites upon annealing because of the dehydration of the samples (Figure 1b). Compared among annealed composites, a-MCN1 maintained the largest *d*-spacing at about 11.70 Å.

**Figure 3:**
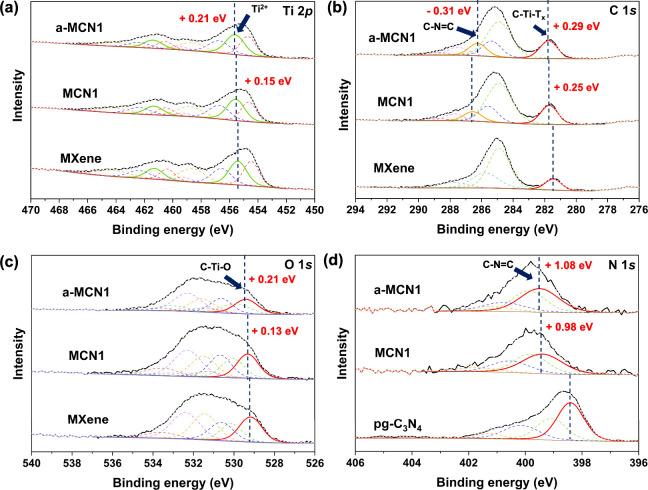
XPS spectra of the freestanding films, as well as relevant energy shifts, for (a) Ti 2p, (b) C 1s, (c) O 1s, and (d) N 1s.

The interfacial interaction of Ti_3_C_2_T_*x*_/pg-C_3_N_4_ was examined by using Raman spectroscopy at an excitation wavelength of 785 nm. For MXene, the observed Raman shift was consistent with the literature (Figure S9a).^59^ The two distinct Raman shifts were located at 200 and 719 cm^–1^, which corresponded to the out-of-plane vibration of surface termination and carbon in Ti_3_C_2_T_*x*_, respectively. After annealing, there was no change in the Raman spectra for a-MXene. Therefore, annealing at this temperature did not appear to alter the surface termination of MXene (Figure S9b). The chemical nature of interfaces within the composites was further investigated by using XPS analysis (see results in Table S1). For Ti 2p, two doublet peaks were observed, which corresponded to Ti 2p_1/2_ and Ti 2p_3/2_ (Figure 3a). For the Ti 2p_3/2_ of the pristine MXene, there were five peaks centering at 454.48, 455.42, 456.58, 458.63, and 459.26 eV that related to Ti^1+^, Ti^2+^, Ti^3+^, TiO_2–*x*_F_2*x*_, and Ti–F, respectively.^60^ After pg-C_3_N_4_ was introduced into Ti_3_C_2_T_*x*_, a shift of +0.15 eV was observed for Ti^2+^, and an additional shift of +0.06 eV occurred upon annealing. This indicates the partial oxidation of MXene when mixed with pg-C_3_N_4_. For the C 1*s*, there were four peaks centering at 281.46, 284.88, 285.78, and 287.81 eV (Figure 3b) that corresponded to C–Ti-T_*x*_, adventitious carbon (C–C), and carbonyl group from ambient hydrocarbon (C–O and C=O), respectively.^60^ The samples were maintained in vacuum-sealed polyethylene bags prior to XPS analysis, which may have introduced microplastic contaminants capable of influencing the C 1s signal. Additionally, during the transfer of the sample from storage to the XPS chamber, there is a possibility of the deposition of adventitious carbon from the surrounding air, forming a thin film on the sample surface. These factors collectively have the potential to compromise the integrity of the C 1s signal, particularly as the film surfaces were not subjected to sputter cleaning prior to data acquisition.^61^

In the case of MCN1, there were four additional peaks located at 285.58, 286.55, 287.71, and 288.85 eV, which could be ascribed to C-NH_*x*_, C–N–C, CO_*x*_, and COOH of pg-C_3_N_4_, respectively.^62−64^ It is interesting to note that upon inclusion of pg-C_3_N_4_, the binding energy of C–Ti-T_*x*_ in Ti_3_C_2_T_*x*_ was shifted by +0.25 eV (Figure 3b), whereas the binding energy of the C–N–C species in pg-C_3_N_4_ was lowered by −1.53 eV (Figure S10a). The annealing process further increased the binding energy by +0.04 eV for C–Ti-T_*x*_ but decreased it by −0.31 eV for pg-C_3_N_4_. For the O 1s, the pristine MXene showed six peaks centering at 529.18, 530.12, 530.62, 531.45, 532.40, and 533.59 eV (Figure 3c), corresponding to the bridge form of oxygen termination (C–Ti–O (i)), TiO_2–*x*_F_2*x*_, face-centered cubic form of oxygen termination (C–Ti–O (ii)), C–Ti–OH, oxygen contamination, and absorbed water, respectively.^65,66^ For pg-C_3_N_4_, there were three peaks at 531.95, 533.53, and 535.19 eV, corresponding to C=O, C–O–C, and C–OH, respectively (Figure S10b).^67^ The oxygen on surface termination showed a significant shift to higher binding energy when pg-C_3_N_4_ was introduced and also upon annealing, while the oxygen species in pg-C_3_N_4_ were barely affected. Thus, the oxygen species in pg-C_3_N_4_ were not directly involved in the interaction with Ti_3_C_2_T_*x*_. For the N 1*s*, the pg-C_3_N_4_ precursor showed four species, including C–N=C, C–NH_2_, C–NH–C, and N–C_3_ at 398.41, 399.09, 400.14, and 401.23 eV, respectively (Figure S10c).^63^ The broad peak at 404.58 eV was assigned as a satellite peak.^62^ All of the peaks in the N 1*s* region shifted to higher binding energies after pg-C_3_N_4_ doping and remained nearly unchanged upon annealing (Figure 3d).

The increase in binding energy for Ti^2+^, as well as carbon atoms and oxygen termination in Ti_3_C_2_T_*x*_, coupled with the decrease in the binding energy related to C–N–C suggest that the charge-storage pathway could be localized in the region formed by the oxygen species of Ti_3_C_2_T_*x*_ and the carbon in the pg-C_3_N_4_ nanosheet. The annealing process that led to better interfacial contact between Ti_3_C_2_T_*x*_ and pg-C_3_N_4_ could also further improve the electron transfer, which was previously observed in the literature.^48^

**Figure 4:**
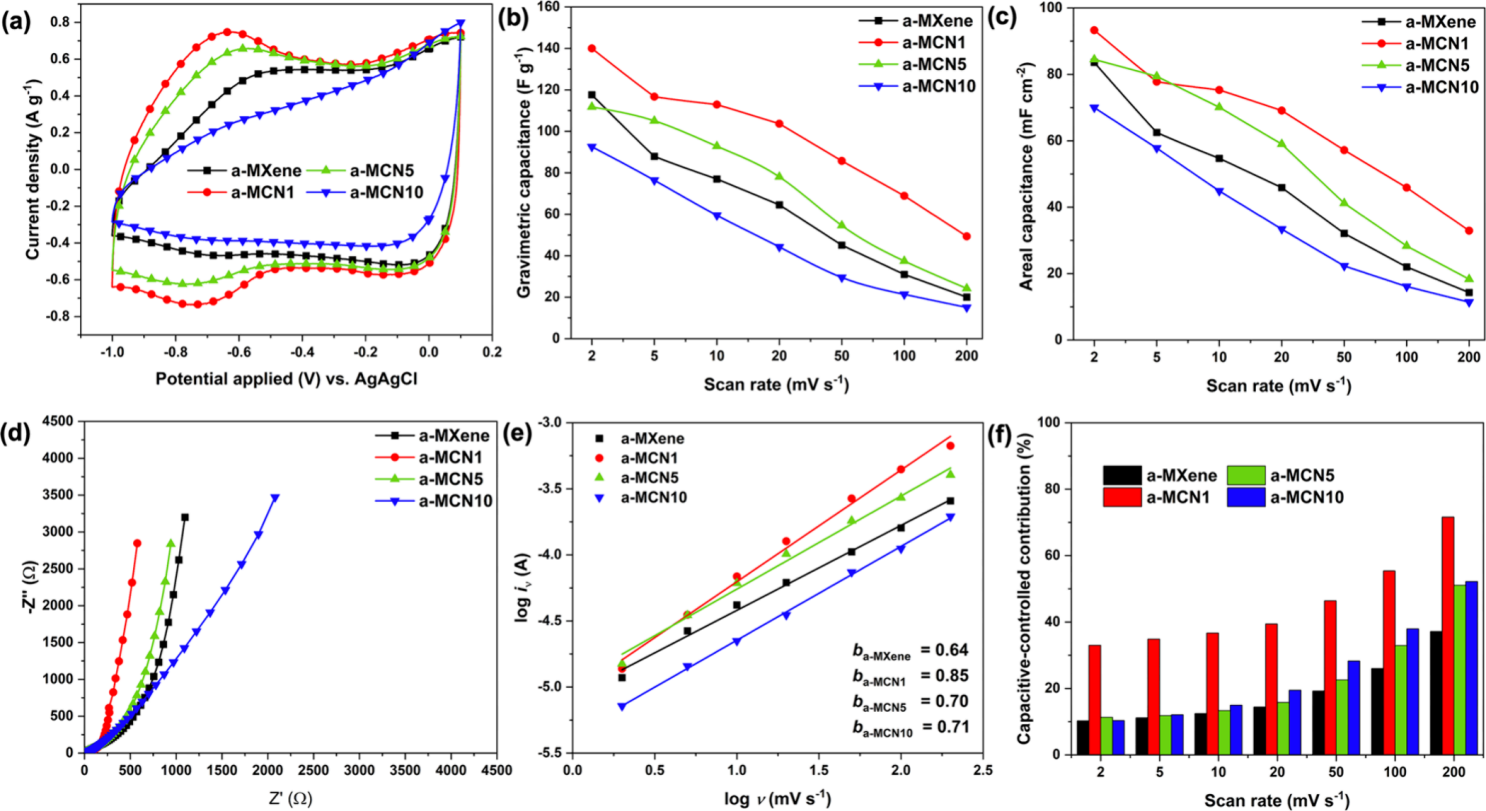
Electrochemical characterization of annealed freestanding films: (a) cyclic voltammogram at 5 mV s^–1^, calculated (b) gravimetric and (c) areal capacitance, (d) EIS spectra, (e) *b* values obtained from the cathodic peak current and potential scan rate, and (f) comparison of capacitive contribution obtained from the Dunn’s method fitting.

Electrochemical measurement of annealed films was performed by using the same conditions as the unannealed samples. The potential window for all samples was stable at 1.1 V after annealing (Figure 4a). a-MCN1 and a-MCN5 showed improved intercalation signal at about −0.65 V, while a-MXene and a-MCN10 showed a reduced signal. Similar to the unannealed samples, a-MCN1 exhibited the highest gravimetric capacitance of about 140 F g^–1^ at 2 mV s^–1^, which was higher than MXene and a-MXene. However, increasing the pg-C_3_N_4_ contents to 5 and 10 wt % resulted in significant drops in gravimetric capacitance to 112 and 93 F g^–1^, respectively (Figure 4b). Overall, the capacitances for most samples were slightly improved upon annealing, except for a-MCN5 and a-MCN10, which can be ascribed to higher degree of porosity as a consequence of annealing.^68,69^ The rate performances of each annealed film were calculated to be 17.1, 35.3, 21.7, and 16.3% for a-MXene, a-MCN1, a-MCN5, and a-MCN10, respectively. The high rate performance for a-MCN1 could arise from its improved electrical conductivity or low charge-transfer resistance as measured using EIS (81.5, 27.2, 50.1, and 63.1 Ω for a-MXene, a-MCN1, a-MCN5, and a-MCN10, respectively, see Figure 4d).

The electrochemical kinetic information indicated an increase in the *b*-value after annealing, which peaked at 0.85 for a-MCN1 (Figure 4e). Similarly, the Dunn’s method analysis revealed the improvement in the capacitive contribution of the composites, especially at high scan rates (Figure 4f). Interestingly, the capacitive contribution for a-MXene was decreased upon annealing, which might be due to narrowed interlayer spacing with a limited diffusion pathway.

**Figure 5:**
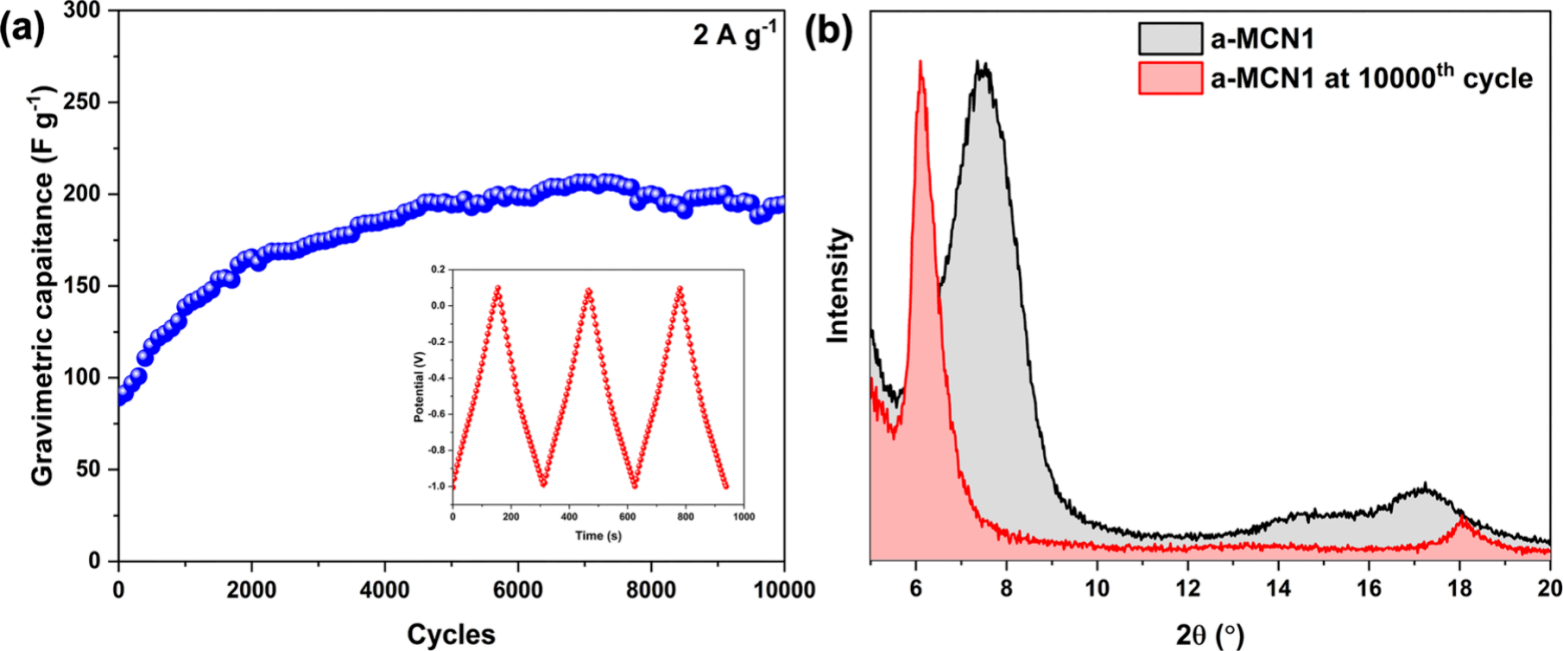
(a) Cycling stability test by galvanostatic charge–discharge at 2 A g^–1^ for 10000 cycles and (b) XRD spectra of a-MCN1 after the 10000th cycle.

The cycling stability test was performed using galvanostatic charge and discharge at 2 A g^–1^ for 10000 cycles (Figure 5a). The composite a-MCN1 showed superior stability with increasing capacitance to 195 F g^–1^ toward the end of the 10000th cycle. The gradual increase in capacitance can be attributed to more accessible ion intercalation sites, which are created during the electrochemical cycling.^19^ Consistent with the previous reports,^19,70^ the XRD analysis also revealed *d*-spacing enlargement after cycling (Figure 5b), which further supports the idea that the electrode possesses more intercalation sites after cycling.

## 3 Conclusions

The Ti_3_C_2_T_*x*_ MXene represents a promising material for high-performance supercapacitors. However, its limited performance in neutral electrolyte systems remains a challenge. One approach that could unlock the limited potential of MXene in neutral electrolytes could be the enhancement of ion diffusion within the electrode material. In this work, we sought to enlarge the *d*-spacing within the MXene structure by introducing pg-C_3_N_4_ via a facile self-assembling process. Indeed, the resulting flexible Ti_3_C_2_T_*x*_/pg-C_3_N_4_ heterostructured material showed increased interlayer spacing, which, in turn, led to a more pronounced fast surface redox contribution, plausibly as a result of better ion diffusion. The annealing process was applied to the Ti_3_C_2_T_*x*_/pg-C_3_N_4_ nanocomposite, which resulted in improved electrical conductivity of up to 8223 S cm^–1^ for the optimal 1 wt % pg-C_3_N_4_ composite. The XPS data suggested a stronger interaction at the O-terminated site of Ti_3_C_2_T_*x*_ surface and reconjugation of g-C_3_N_4_ after annealing. The measured operating potential was stable within the range of 0.1 to 1.0 V. At the optimal 1 composition in Ti_3_C_2_T_*x*_, the gravimetric capacitance of 140 F g^–1^ was obtained. The cycling stability measurement indicated excellent capacitance retention during 10000 charge–discharge cycles, at which the gravimetric capacitance was increased up to 195 F g^–1^.

## Supporting Information Available

Experimental details, X-ray diffractograms of precursors, UV–vis spectrum of delaminated Ti_3_C_2_T_*x*_ solution, AFM image of Ti_3_C_2_T_*x*_ single layer, physical appearance of g-C_3_N_4_ powder, IR spectra of synthesized g-C_3_N_4_ and pg-C_3_N_4_, particle size distribution, SEM top-view images of g-C_3_N_4_ powder and cross-sectional image of all prepared films, equivalent circuit model for EIS fitting, Raman spectra, XPS peak fitting data for a-MCN1, XPS spectra of synthesized pg-C_3_N_4_, and comparison potential window and capacitance with other literature studies (PDF)
