# Supplementary material for: Interfacial Engineering of Ti3C2Tx MXene Electrode Using g-C3N4 Nanosheets for High-Performance Supercapacitor in Neutral Electrolyte
Source: ACS Omega. 2024 May 6;9(20):22256–64. doi: 10.1021/acsomega.4c01353 (PMC11112722; doi:10.1021/acsomega.4c01353)
Supplement: Supplementary file 1 — ao4c01353_si_001.pdf [file ao4c01353_si_001.pdf]

Supporting Information

Interfacial engineering of $Ti_3C_2T_x$ MXene electrode using g-C_3N_4 nanosheets for high performance supercapacitor in neutral electrolyte

Manopat Depijan[1], Kanit Hantanasirisakul[2], and Pasit Pakawatpanurut[1,]*

[1]Department of Chemistry, Center of Excellence for Innovation in Chemistry, and Center of Sustainable Energy and Green Materials, Faculty of Science, Mahidol University, 272 Rama VI Road, Ratchathewi, Bangkok 10400, Thailand

[2]Centre of Excellence for Energy Storage Technology (CEST), Department of Chemical and Biomolecular Engineering, School of Energy Science and Engineering, Vidyasirimedhi Institute of Science and Technology, Wangchan Valley, Rayong 21210, Thailand

*Corresponding author E-mail: pasit.pk@gmail.com

Experimental section

Synthesis of Delamination $Ti_3C_2T_x$

$Ti_3C_2T_x$ was synthesized following the minimally intensive layer delamination (MILD) method.[1] Briefly, 1.6 g of lithium fluoride)LiF, >98% purity, Alfa Aesar(was dissolved in 20 mL of 9 M HCl (RCI Labscan, Ltd.) solution and stirred for 5 min in an HDPE bottle. This solution was then moved to an ice bath, and 1 g of Ti_3AlC_2 (MAX) powder (>99.5%, >500 mesh, Laizhou Kai Kai Ceramic Materials Co., Ltd.) was slowly added to the solution. Then, the resulting mixture was stirred at 35 °C for 24 h. The mixture was washed several times with deionized water via repeated centrifugation at 3500 rpm until the pH was neutral. After that, the mixture was shaken manually for 10 min for further exfoliation, followed by centrifugation for 1 h at 3500 rpm. The supernatant was extracted and stored in a refrigerator. The concentration of the colloidal solution was kept at 0.5 mg mL^{-1}.

Synthesis of Protonated Graphitic Carbon Nitride (pg-C_3N_4) Nanosheet

Protonated g-C_3N_4 was synthesized according to the literature.[2] Briefly, 1 g of dicyandiamide powder (99%, Sigma Aldrich) and 10 g of ammonium chloride were mixed and then heated in air at 550 °C for 4 h. The obtained pale-yellow powder was then sonicated in ethanol for 2 h. To ensure complete delamination of the nanosheets, 100 mL concentrated H_2SO_4 (98% RCI Labscan, Ltd) was added, and the mixture was vigorously stirred at room temperature for 12 h. Then, the obtained milk-like solution was washed with deionized water via vacuum filtration. The protonated g-C_3N_4 nanosheet (pg-C_3N_4) was dispersed in deionized water under sonication for 30 min, resulting in a stably dispersed pg-C_3N_4 solution. The concentration was kept at 0.02 mg mL^{-1}.

Fabrication of $Ti_3C_2T_x$/pg-C_3N_4 Freestanding Film

The composited freestanding films were fabricated via the self-assembling process. The prepared $Ti_3C_2T_x$ colloidal solution was gradually added into pg-C_3N_4 solution and stirred for 30 min using varying pg-C_3N_4 contents from 1 wt.% to 10 wt.%. During this step, the negatively charged MXene sheets self-assembled with the positively-charged pg-C_3N_4 in solution. The mixture was then vacuum-filtrated through a hydrophilic PVDF membrane (47 mm, pore size 0.22 μm). After drying, the freestanding film of $Ti_3C_2T_x$/pg-C_3N_4 was peeled off from the membrane. The $Ti_3C_2T_x$/pg-C_3N_4 films prepared with 0, 1, 5, and 10 wt.% pg-C_3N_4 were denoted as MXene, MCN1, MCN5, and MCN10, respectively. The as-prepared freestanding electrode has a mass loading about 1.14 mg cm^{-2}. To improve the conductivity and interfacial contact of the composite, the prepared freestanding film was annealed at 200 °C for 2 h under an Ar atmosphere. The annealed samples were labeled as a-MXene, a-MCN1, a-MCN5, and a-MCN10.

Materials Characterization

X-ray diffractometer (XRD, PANalytical EMPYREAN, Cu Kα radiation 1.542 Å) was carried out to investigate the sample crystallography at 2θ = 5–70 at 2° min^{-1} of scan rate. A Cold field emission scanning electron microscope (FE-SEM, SU8010, Hitachi(and energy-dispersive X-ray analyzer (EDX) was utilized to obtain cross-sectional images and element mapping. A Zetasizer (Malvern instrument, Nanoseries ZS, Worcestershire, UK) was used for zeta potential and particle size distribution measurements. A Fourier-transform infrared spectrometer (FTIR, PerkinElmer 2000) was used for the characterization of the surface functional group. A UV-visible spectrophotometer (UV-VIS, Shimadzu UV-2600) was employed to measure the optical properties of the colloidal $Ti_3C_2T_x$ solution, and the solution

concentration was deduced from the absorbance at 760 nm. Atomic force microscope (AFM) (NX 10, Park Systems, Korea) was utilized for the thickness measurement of MXene single flake. Raman spectroscopy (Thermo Fisher Scientific, DXR smart Raman, 785 nm excitation laser) was used to study material interaction. The valence states of the elements were investigated using X-ray photoelectron spectroscopy (XPS) with a Kratos AMICUS instrument, employing an Mg Kα anode (1253.6 eV, 10 mA, 10 kV). The X-ray incidence angle on the sample was set to 50°, with an emission angle of 90°. The base pressure was maintained below 10^{-8} Pa. Samples were cut into 6 mm circular films and fully loaded into the sample holder, with an analysis area of approximately 200 μm in beam diameter. Due to the highly conductive nature of our samples and the absence of sputtering during measurement, a charge neutralizer was not utilized. Prior to analysis, all samples were stored in vacuum-sealed polyethylene bags. The full width at half maximum (FWHM) of the Ag $3d_{5/2}$ peak was approximately 1 eV. Binding energies were calibrated using the C1s (C-C) peak of adventitious carbon at 284.88 eV. The reference binding energy (E_B^F) was calculated as E_B^F = 289.98 eV + ϕ_{SA}, where ϕ_{SA} represented the material's work function energy, approximately 4.7 eV.[3,4] XPS spectra were analyzed using CasaXPS software, employing Shirley background subtraction and asymmetric line shape fitting.

Electrochemical Characterization

All electrochemical measurements were performed in a three-electrode Swagelok cell. The prepared freestanding film of $Ti_3C_2T_x$/pg-C_3N_4, activated carbon freestanding film (Kuraray YP-50F), Ag/AgCl in 3 M KCl, and 1 M $MgSO_4$ served as working electrode, counter electrode, reference electrode, and electrolyte, respectively. Cyclic voltammogram (CV) was measured from -1.0 V to 0.1 V with the scan rate from 2 to 200 mV s^{-1}. In three-electrode system, gravimetric (C_g) and areal capacitances (C_A) were calculated from the CV discharging curves

using eq 1 and eq 2, where i, V, m, v, ΔV, A denote current (A), potential applied (V), mass of the freestanding film (g), scan rate (V s^{-1}), potential window (V), and the area of electrode (cm^2), respectively.

$$C_g = \frac{\int idV}{mv\Delta V}, \qquad (1)$$

$$C_A = C_g \times \frac{m}{A} \qquad (2)$$

The cyclic galvanostatic charge-discharge (GCD) was carried out for stability measurement. The electrochemical impedance spectroscopy (EIS) was used for impedance measurement with 10 mV amplitude in the range of 10 mHz to 100 kHz.

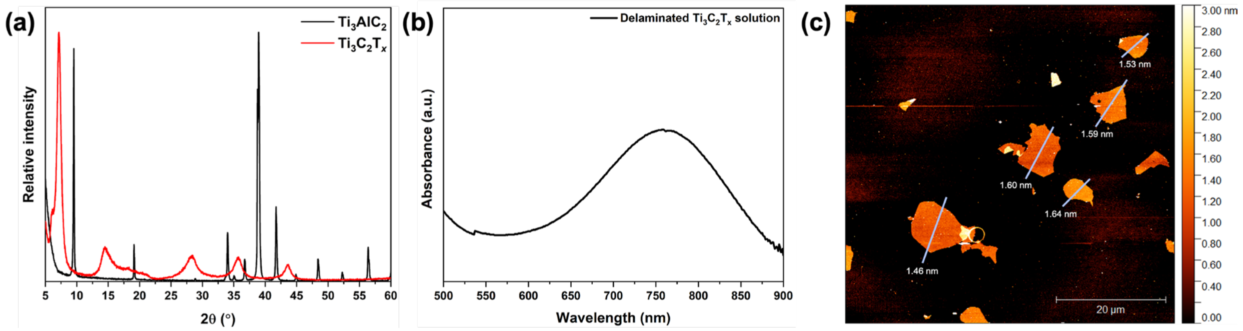

Figure S1. (a) The X-ray diffractograms of Ti_3AlC_2 compared with $Ti_3C_2T_x$ after etching, (b) UV-vis spectrum of delaminated $Ti_3C_2T_x$ solution, and (c) AFM image of the $Ti_3C_2T_x$ single layers.

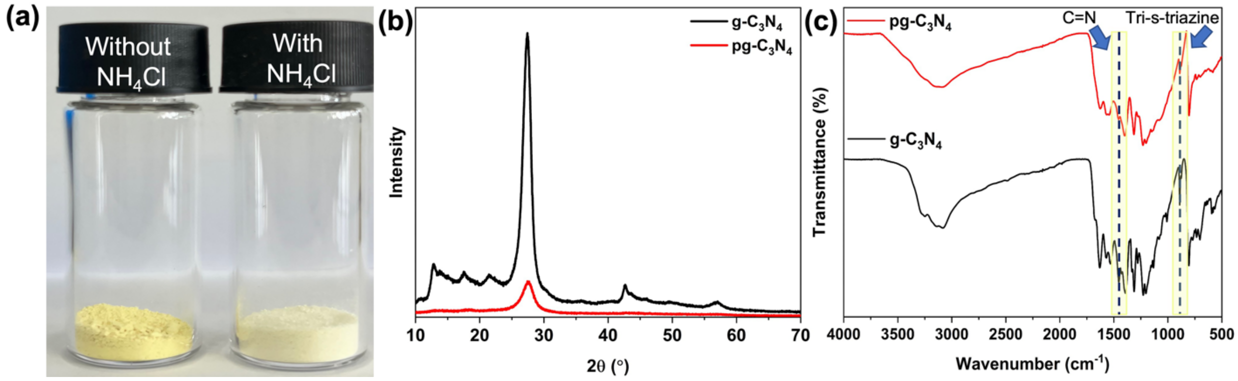

Figure S2. (a) $g-C_3N_4$ powder obtained after sintering at 550 °C for 4 h without ammonium chloride (left) and with ammonium chloride (right), and the comparison of (b) X-ray diffractogram and (c) IR spectra of synthesized $g-C_3N_4$ and $pg-C_3N_4$.

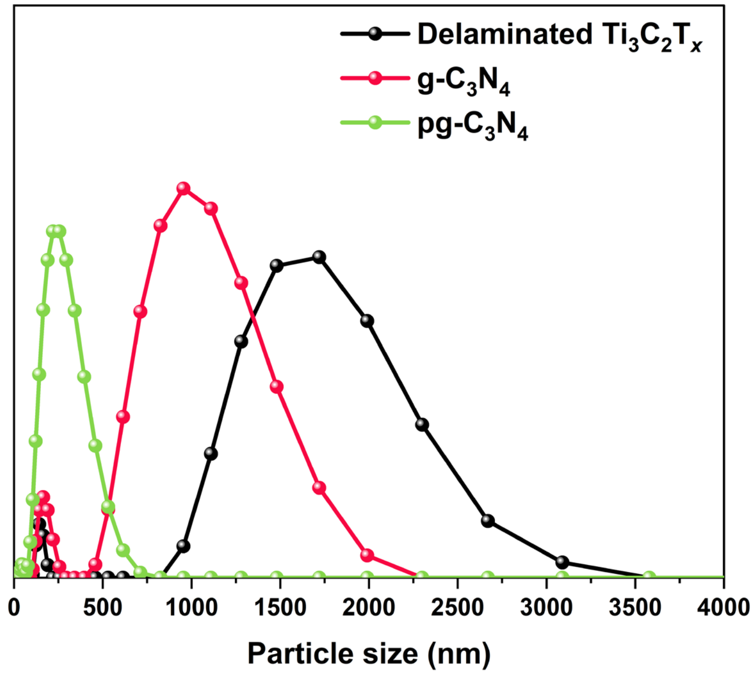

Figure S3. Particle size distribution of the prepared precursors in water.

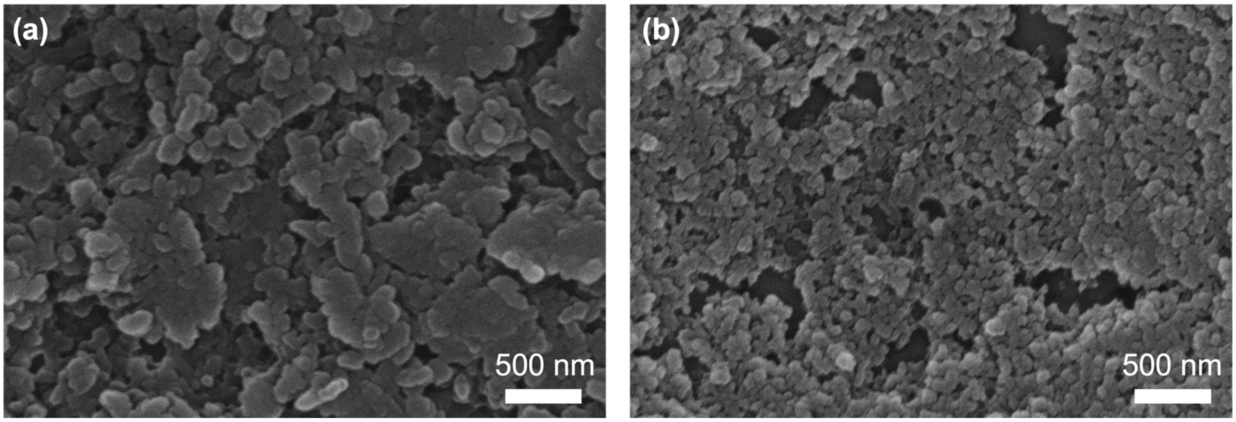

Figure S4. Top-view SEM images of (a) unprotonated g-C_3N_4 and (b) protonated g-C_3N_4.

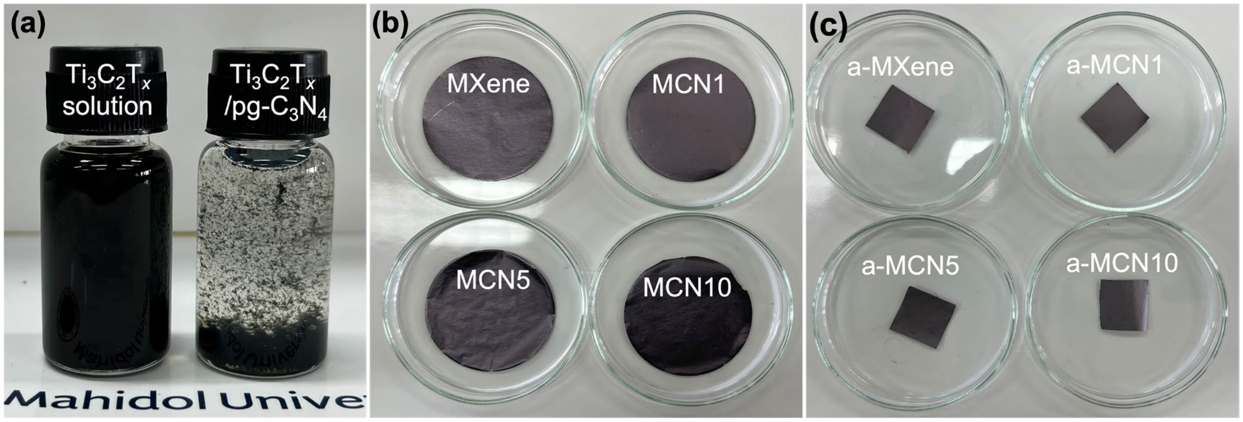

Figure S5. (a) The delaminated $Ti_3C_2T_x$ suspension in water before and after being mixed with pg-C_3N_4, and $Ti_3C_2T_x$/pg-C_3N_4 freestanding film (b) before and (c) after annealing.

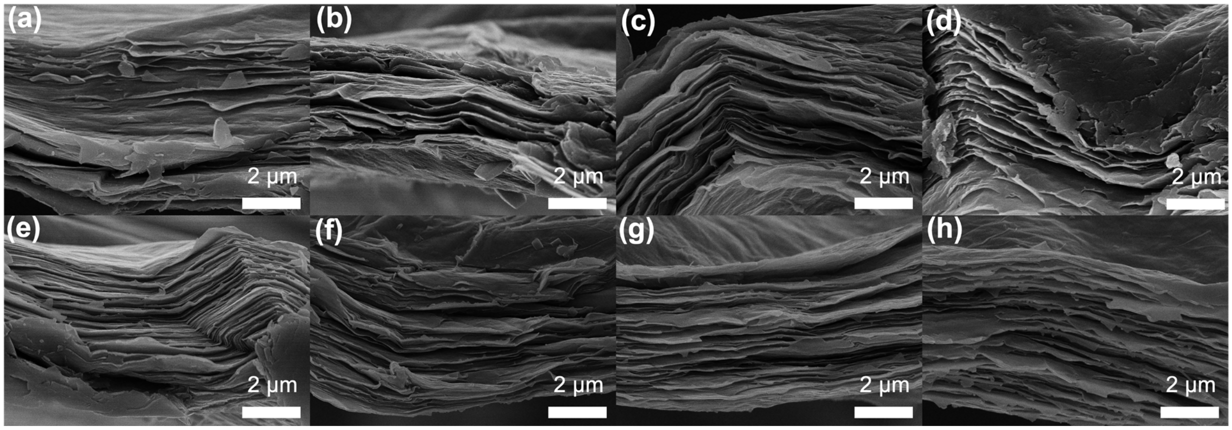

Figure S6. Cross-sectional images of freestanding films of (a) MXene, (b) MCN1, (c) MCN5, (d) MCN10, (e) a-MXene, (f) a-MCN1, (g) a-MCN5, and (h) a-MCN10.

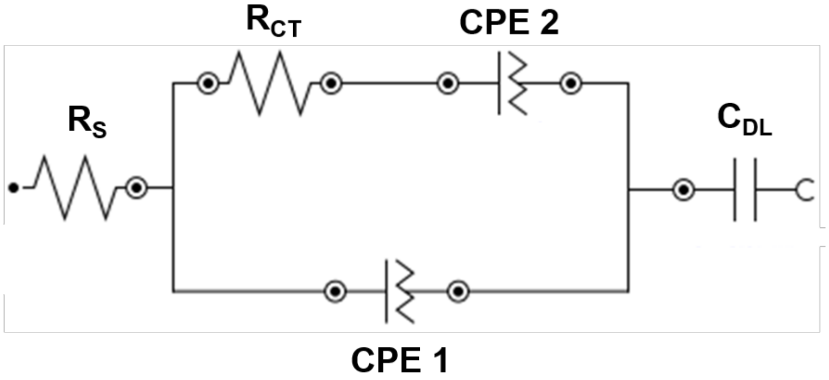

Figure S7. Equivalent circuit model for EIS fitting, where R_s, R_{CT}, CPE, and C_{DL} corresponding to electrode resistance, charge-transfer resistance, constant phase element, and double-layer capacitance, respectively.

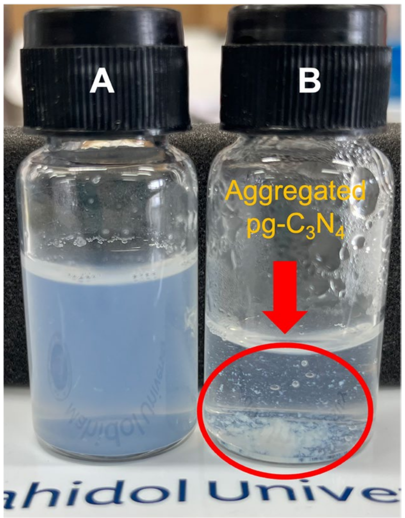

Figure S8. Physical appearance of pg-C_3N_4 in water (a) before and (b) after thermal treatment.

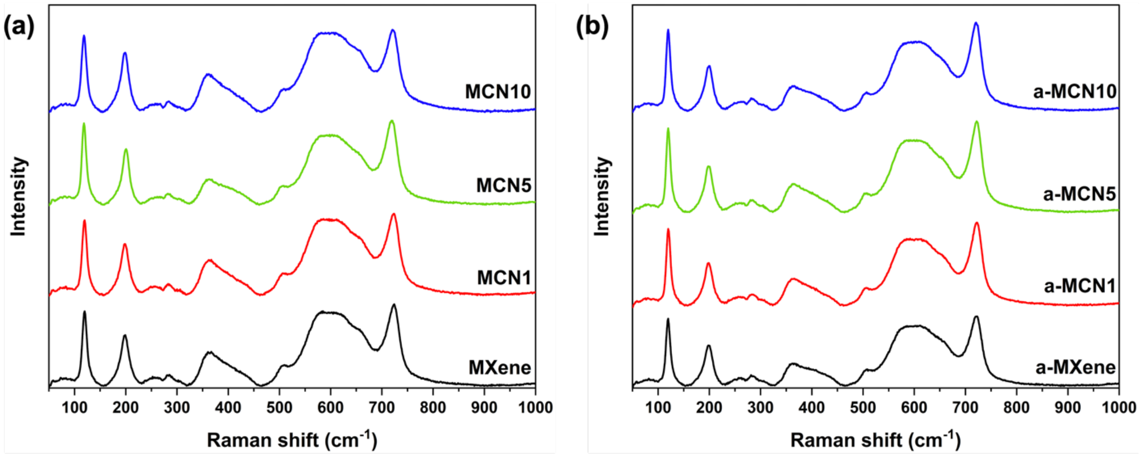

Figure S9. Raman spectra at 785 nm excitation wavelength of the freestanding films (a) before and (b) after annealing.

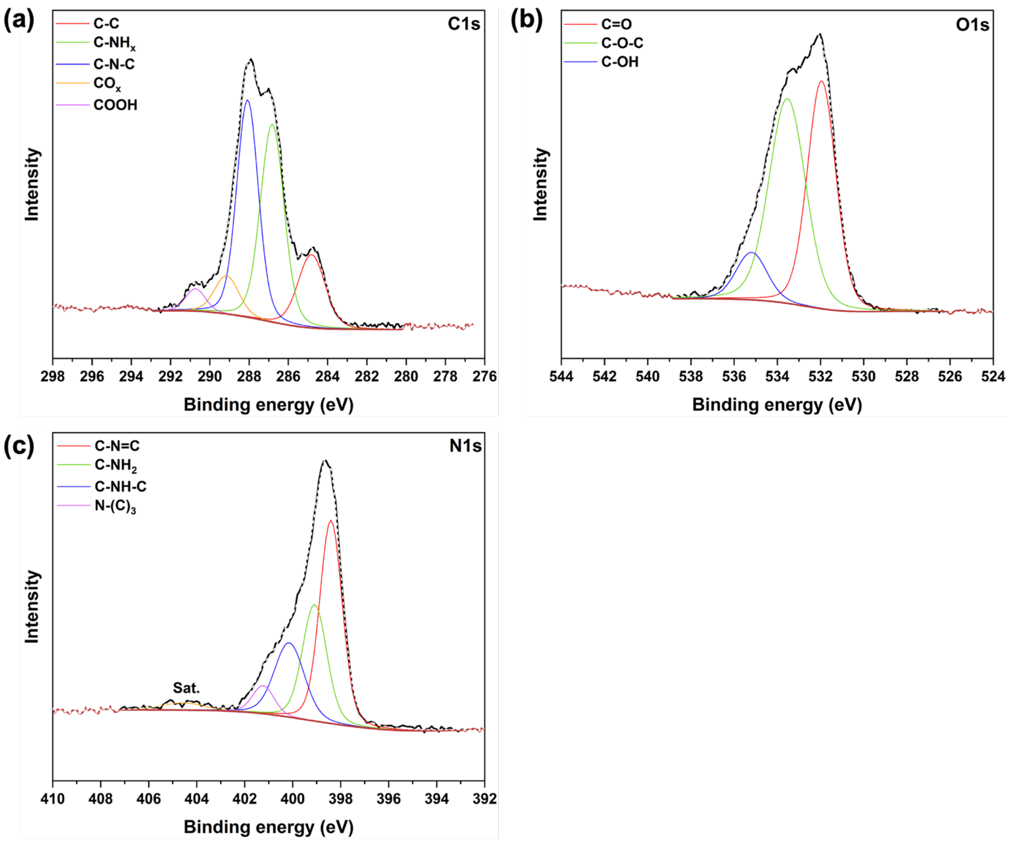

Figure S10. XPS spectra of the as-synthesized pg-C_3N_4 for (a) C 1s, (b) O 1s, and (c) N 1s.

Table S1. XPS peak fitting data for a-MCN1, consisting of peak position, FWHM, and the definition of each species.

Species	Region	BE (eV)	FWHM	Definition
**Ti^{+1} $2p_{3/2}$ ($2p_{1/2}$)**	Ti 2p	454.72 (460.54)	1.28 (1.27)	Ti atoms bounded with surface terminations
**Ti^{+2} $2p_{3/2}$ ($2p_{1/2}$)**		455.63 (461.38)	1.55 (1.50)	
**Ti^{+3} $2p_{3/2}$ ($2p_{1/2}$)**		456.75 (462.38)	1.76 (1.75)	
**$TiO_{2-x}F_{2x}$ $2p_{3/2}$ ($2p_{1/2}$)**		458.64 (464.7)	2.09 (2.88)	Partially oxidized surface
**Ti-F $2p_{3/2}$ ($2p_{1/2}$)**		459.41 (465.71)	1.15 (1.86)	Secondary phase of F termination
**C-Ti-T_x**	C 1s	281.75	1.32	C within MXene
**C-C**		284.88	1.85	Adventitious carbon
**C-NH_X**		285.37	1.42	C from g-C_3N_4
**C-N-C**		286.24	1.32	
**CO_x**		287.51	1.49	Oxidized g-C_3N_4
**COOH**		288.82	1.38	
**C-Ti-O**	O 1s	529.39	1.36	Bridge form of O termination
**$TiO_{2-x}F_{2x}$**		529.90	1.62	$TiO_{2-x}F_{2x}$
**C-Ti-O (ii)**		530.62	1.23	FCC form of O termination
**C-Ti-OH**		531.57	1.44	Hydroxyl group
**OR**		532.28	1.65	Contamination
**H_2O**		533.57	1.49	Adsorbed water
**C=O**		531.36	1.58	Oxidized g-C_3N_4
**C-O-C**		533.07	1.85	
**C-OH**		534.9	1.70	

**C-Ti-F**	F 1s	684.66	1.65	F from MXene
**F contamination**		686.09	1.65	Unknown F contamination
**F contamination**		687.61	2.32	
**Cl $2p_{3/2}$ ($2p_{1/2}$)**	Cl 2p	199.08 (200.69)	1.27(2.19)	Cl surface termination
**C-N=C**	N 1s	399.49	1.59	N from g-C_3N_4
**C-NH_2**		399.87	1.35	
**C-NH-C**		400.75	1.73	
**N-C_3**		401.23	1.80	

**Species**	Region	BE (eV)	FWHM	Definition
**C-NH_2**		400.42	1.4	
**C-NH-C**		401.13	1.62	
**N-C_3**		402.37	1.75	

Table S2 Comparison of potential window and capacitance between this work and previous reports.

Electrode Material	Electrolyte	Capacitance	Potential window (V)	Reference
DMSO-$Ti_3C_2T_x$	$MgSO_4$	96 F g^{-1} @ 2 mV s^{-1}	0.6	5
DMSO-$Ti_3C_2T_x$ freestanding film	$MgSO_4$	100 F g^{-1} @ 2 mV s^{-1}	0.8	5
$Ti_3C_2T_x$/SWCNT	$MgSO_4$	102 F g^{-1} @ 2 mV s^{-1}	0.9	6
$Ti_3C_2T_x$/MWCNT	$MgSO_4$	127 F g^{-1} @ 2 mV s^{-1}	0.9	6
$Ti_3C_2T_x$/rGO	$MgSO_4$	435 F cm^{-3} @ 2 mV s^{-1}	0.9	6
N-doped multilayer $Ti_3C_2T_x$	$MgSO_4$	82 F g^{-1} @ 1 mV s^{-1}	0.6	7
TMAOH-$Ti_3C_2T_x$ freestanding film	$MgSO_4$	176 F g^{-1} @ 0.5 mV s^{-1}	0.9	8
TMAOH-$Ti_3C_2T_x$ freestanding film	Na_2SO_4	134 F g^{-1} @ 0.5 mV s^{-1}	0.9	8
TMAOH-$Ti_3C_2T_x$ freestanding film	Li_2SO_4	150 F g^{-1} @ 0.5 mV s^{-1}	0.9	8
TMAOH-$Ti_3C_2T_x$ freestanding film	K_2SO_4	127 F g^{-1} @ 0.5 mV s^{-1}	0.9	8
$Ti_3C_2T_x$	$MgSO_4$	75 F g^{-1} @ 2 mV s^{-1}	0.4	9
Annealed $Ti_3C_2T_x$ freestanding film	$MgSO_4$	118 F g^{-1} @ 2 mV s^{-1}	1.1	This work
Protonated g-C_3N_4 doped $Ti_3C_2T_x$ freestanding film	$MgSO_4$	140 F g^{-1} @ 2 mV s^{-1}	1.1	This work

REFERENCES

(1) Alhabeb, M.; Maleski, K.; Anasori, B.; Lelyukh, P.; Clark, L.; Sin, S.; Gogotsi, Y. Guidelines for Synthesis and Processing of Two-Dimensional Titanium Carbide (Ti 3 C 2 T x MXene). *Chem. Mater.* **2017**, *29* (18), 7633–7644. https://doi.org/10.1021/acs.chemmater.7b02847.

(2) Lu, X.; Xu, K.; Chen, P.; Jia, K.; Liu, S.; Wu, C. Facile One Step Method Realizing Scalable Production of G-C 3 N 4 Nanosheets and Study of Their Photocatalytic H 2 Evolution Activity. *J. Mater. Chem. A* **2014**, *2* (44), 18924–18928. https://doi.org/10.1039/c4ta04487h.

(3) Schultz, T.; Frey, N. C.; Hantanasirisakul, K.; Park, S.; May, S. J.; Shenoy, V. B.; Gogotsi, Y.; Koch, N. Surface Termination Dependent Work Function and Electronic Properties of Ti3C2T x MXene. *Chem. Mater.* **2019**, *31* (17), 6590–6597. https://doi.org/10.1021/acs.chemmater.9b00414.

(4) Greczynski, G.; Hultman, L. Reliable Determination of Chemical State in X-Ray Photoelectron Spectroscopy Based on Sample-Work-Function Referencing to Adventitious Carbon: Resolving the Myth of Apparent Constant Binding Energy of the C 1s Peak. *Appl. Surf. Sci.* **2018**, *451*, 99–103. https://doi.org/10.1016/j.apsusc.2018.04.226.

(5) Lukatskaya, M. R.; Mashtalir, O.; Ren, C. E.; Dall'Agnese, Y.; Rozier, P.; Taberna, P. L.; Naguib, M.; Simon, P.; Barsoum, M. W.; Gogotsi, Y. Cation Intercalation and High Volumetric Capacitance of Two-Dimensional Titanium Carbide. *Science* **2013**, *341* (6153), 1502–1505. https://doi.org/10.1126/science.1241488.

(6) Zhao, M.; Ren, C. E.; Ling, Z.; Lukatskaya, M. R.; Zhang, C.; Aken, K. L. V.; Barsoum, M. W.; Gogotsi, Y. Flexible MXene/Carbon Nanotube Composite Paper with High Volumetric Capacitance. *Adv. Mater.* **2015**, *27* (2), 339–345. https://doi.org/10.1002/adma.201404140.

(7) Wen, Y.; Rufford, T. E.; Chen, X.; Li, N.; Lyu, M.; Dai, L.; Wang, L. Nitrogen-Doped Ti3C2Tx MXene Electrodes for High-Performance Supercapacitors. *Nano Energy* **2017**, *38*, 368–376. https://doi.org/10.1016/j.nanoen.2017.06.009.

(8) Ren, S.; Xu, J.-L.; Cheng, L.; Gao, X.; Wang, S.-D. Amine-Assisted Delaminated 2D Ti3C2T x MXenes for High Specific Capacitance in Neutral Aqueous Electrolytes. *ACS Appl. Mater. Inter.* **2021**, *13* (30), 35878–35888. https://doi.org/10.1021/acsami.1c06161.

(9) Gao, Z.; Zheng, W.; Lee, L. Y. S. Highly Enhanced Pseudocapacitive Performance of Vanadium-Doped MXenes in Neutral Electrolytes. *Small* **2019**, *15* (40), 1902649. https://doi.org/10.1002/smll.201902649.
